# Bilateral and Unilateral Asymmetries of Isokinetic Strength and Flexibility in Male Young Professional Soccer Players

**DOI:** 10.2478/hukin-2013-0005

**Published:** 2013-03-28

**Authors:** Abdolhamid Daneshjoo, Nader Rahnama, Abdul Halim Mokhtar, Ashril Yusof

**Affiliations:** 1 Sports Centre, University of Malaya, Kuala Lumpur, Malaysia.; 2 Faculty of Physical Education and Sport Science, University of Isfahan, Isfahan, Iran.; 3 Faculty of Medicine, University of Malaya, Kuala Lumpur, Malaysia.

**Keywords:** isokinetic strength, flexibility, competitive soccer players

## Abstract

This study investigated bilateral and unilateral asymmetries of strength and flexibility in male young professional soccer players. Thirty-six soccer players (age: 18.9 ± 1.4 years) participated in this study. A Biodex Isokinetic Dynamometer was used to assess the hamstring and quadriceps strength at selected speeds of 60°/s, 180°/s and 300°/s. Hip joint flexibility was measured using a goniometer. No difference was observed in conventional strength ratio, dynamic control ratio and fast/slow speed ratio between the dominant and non-dominant legs (p>0.05). All but one of the players (97.2%) had musculoskeletal abnormality (bilateral imbalance > 10%) in one or more specific muscle groups. The dominant leg had greater hip joint flexibility compared with the non-dominant leg (108.8 ± 10.7° versus 104.6 ± 9.8°, respectively). The findings support the hypothesis that physical performance and movement pattern experienced during soccer playing may negatively change the balance of strength in both legs (bilateral strength balance), but not on the same leg of the young male professional soccer players. The results can be helpful for trainers and coaches to decide whether the players need to improve their balance and strength which in turn may prevent injury. It is suggested that in professional soccer training, quadriceps and hamstrings muscle strength, as well as hip joint flexibility should not be overlooked.

## Introduction

Strength and flexibility are two of the key indicators of physical performance in soccer players (Lehance et al., 2009). Particularly during dynamic movements in soccer, balance in strength and flexibility between the dominant and non-dominant legs provide joint stability. Strength and flexibility asymmetries between the two limbs and reciprocal strength ratio between the agonist and antagonist muscles especially in lower body reportedly play an important role in sports with asymmetric kinetic patterns like soccer (Rahnama et al., 2005; Fousekis et al., 2010). Most soccer players favour or are forced to use one particularleg for ball kicking and cutting skill (Fousekis et al., 2010) and this preference is the possible cause of asymmetry in the flexibility and strength of the lower extremities between the two legs or between the agonist and antagonist muscles. It is, however, hard to know which agonist or the antagonist muscles in the legs are stronger or weaker. The reason is when a soccer player kicks a ball using the dominant extremity, the other leg is used to support the body weight (Oshita and Yano, 2010).

Strength and flexibility asymmetry of joints or extremities can lead to improper control of body movement (Grygorowicz et al., 2010). One of the first studies in this field was carried out by Knapik et al. (1991). The athletes in their study reported a higher frequency of lower extremity injuries when the right hamstrings were 15% stronger than the left hamstrings at 180°/s, while the right hip extensor was 15% more flexible than the left hip extensor and knee ratio of less than 0.75 at 180°/s. These results revealed that strength and flexibility asymmetries were negatively associated with lower body injuries in female collegiate athletes. Rahnama et al. (2005) reported that knee flexor muscle strength in the non-dominant leg was more than that of the dominant leg in more than 68% English amateur soccer players. In contrast, Brito et al. (2010) found greater peak torque (PT) in the knee flexors and extensors in the dominant leg than the non-dominant leg in sub-elite male soccer players. These conflicting results necessitate further investigation in this area.

Hamstring to quadriceps strength ratio (Hcon/Qcon) between knee extensors and flexors are implicated as an important factor in the predictions of knee function and injuries (Kim and Hong, 2011). Researchers across many sports regard the value of 0.6 as normative for the conventional strength ratio (CSR) at 60°/s which increases up to 0.8 with increased movement velocity (Hewett et al., 2008; Grygorowicz et al., 2010; Hadzic et al., 2010). It has been suggested that the CSR of less than 0.6 would indicate a strength imbalance between the quadriceps and hamstrings which could predispose one to injury (Tourny-Chollet et al., 2000). The results of such testing which used the CSR and dynamic control ratio (DCR= eccentric PT of hamstrings/concentric PT of quadriceps) to describe muscle balance can be used in knee injury prevention (Grygorowicz et al., 2010; Fousekis et al., 2011). It is estimated that there are 270 million soccer players in the world, 90% of whom are male. Out of this population, young players constitute the majority (54.7%) of the registered male players (FIFA, 2006; Alentorn-Geli et al., 2009). In such a large sports population, injury prevention is important in providing healthcare, enhancing health, as well as decreasing costs for medical care in soccer players (Kiani et al., 2010; Rahnama, 2011).

It is generally accepted that increased flexibility enhances sports performance. Athletes with a high degree of flexibility traditionally present improved proficiency in movements. Flexibility is set by the range of motion (ROM) available at a joint. Flexibility asymmetry is an important internal risk factor for knee injuries. It has been found that low hip flexibility was associated with an increased risk of injury in soccer players (Arnason et al., 2004; Hrysomallis, 2009). At the same time, flexibility throughout the full ROM must be aptly supported by muscles (Bradley and Portas, 2007; Ozcaldiran, 2008).

In brief, the reviewed studies which aimed to analyse PT and strength ratio in athletes, have shown contradictory results. Additionally, little attention has been paid to the muscle PT, flexibility and strength ratio of male professional soccer players. To our knowledge, only few studies have investigated asymmetry of the knee on professional male soccer players. With respect to the increasing knee injuries in young professional soccer players in the world and relationship between strength deficit, strength ratio and flexibility with knee injuries, the main purpose of the present study was to investigate the bilateral and unilateral asymmetries of strength and flexibility in young male professional soccer players.

## Material and Methods

### Participants

Thirty-six male young professional soccer players (age: 18.9 ± 1.4 years; body height: 181.3 ± 5.5 cm; body mass: 73.6 ± 6.3 kg) who had played for at least five years, had been regularly training five sessions per week and also had no history of major lower limb injury or disease participated in this study. The participants were selected from three professional teams. Seventeen players had the experience of playing in the national team. All participants were outfield players and members of a professional football club. During the 2011 competitive season, all players participated in official league championships (First Division). The subjects were orally informed about the procedures they would undergo, and each read and signed an informed consent form. The players were chosen by one of the researchers. The study was approved by the Institute of Research Management and Monitoring, University of Malaya and the Sports Centre Research Committee.

### Isokinetic test

A Biodex Isokinetic Dynamometer (Biodex Medical Systems, Inc., Shirley, New York, USA) was used to assess the hamstring and quadriceps strength of the subjects. Before each testing session, the dynamometer was gravitationally corrected in accordance with the manufacturer’s recommendations. The subjects performed a general cardiovascular warm-up for at least 5 minutes on a Monark cycle ergometer at a moderate pace and load (50–100 W), followed by 10-minute dynamic stretching concentrating on the lower limbs to prepare the subjects for the assessment of leg torque (Hadzic et al., 2010).

Each subject was seated on the chair and assumed the optimal position to enable the researchers to achieve reliable test results. The subjects were secured with snug straps across the shoulder, chest and hip to limit excessive movement. The cuff of the dynamometer lever arm was attached to proximal malleoli of the ankle. Dynamometer orientation was fixed at 90° and tilted at 0°, while the seat orientation was fixed at 90° and the seatback tilted at 75°–85°. All the seating positions of the subjects were recorded carefully and repeated during post-test. The subjects were instructed to complete 3 trials, two sub-maximal efforts and one maximal effort on the isokinetic machine. The subjects then performed 3 repetitions of knee extension and flexion at each selected angular velocity with a 5 s rest interval in between. They were also given a 1 min rest between different angular velocities and a 3 min break when the machine setting was changed for the opposite leg. The order of testing was randomized for the dominant and non-dominant legs. Encouragements by verbal coaching and visual feedback were given to all subjects to help them concentrate on the quality of their movements. All isokinetic contractions performed on the dynamometer at a speed of 60°/s (slow velocity), 180°/s (medium velocity) and 300°/s (high velocity), through a knee range of motion of 0° (flexed) to 90° (full extension) (Masuda et al., 2005; Zakas, 2006; Fousekis et al., 2010). The conventional strength ratio (CSR) was evaluated by concentric PT of hamstrings divided by concentric PT of quadriceps at each angular velocity. The PT (Newton meter) values were recorded for comparison.

### Hip flexibility

The flexibility of the participants’ hip joint was measured using a standard goniometer (ISOM 12″, Baseline NY) according to the method described by Norkin et al. (2003). The subjects were laid on a flat surface in a supine position with legs extended. The axis of the goniometer was placed at the greater trochanter of the femur. A strap was used around the pelvis to minimize lumbosacral movement which may have influenced the results. The right leg was passively lifted to the maximum range by the examiner while keeping the knee of the left leg on the flat surface. The examiner also allowed the knee of the right leg to flex passively during the motion. The right leg was kept fixed at maximum hip flexion and the angle between the moveable arm of goniometer and leg was recorded for hip flexibility. The reading was recorded when tightness was felt by the subject and concurred by the examiner. The average of three attempts was used for analysis. This test was performed on the opposite leg to assess the flexibility of both legs (Rahnama et al., 2005; Manning and Hudson, 2009).

### Musculoskeletal abnormality

In this study, musculoskeletal abnormality of knee muscles was defined as a bilateral strength imbalance of more than 10% (Dauty et al., 2003; Rahnama et al., 2005).

### Statistical analysis

For data analysis and to compare the PT and CSR among velocities (60°/s, 180°/s, 300°/s) and legs (dominant leg, non-dominant leg) a 3 × 2 (speed vs leg), repeated measures ANOVA was used. To compare DCR and flexibility between the dominant and non-dominant legs, a paired sample *t*-test was used. The single *chi*-square statistic was calculated to compare the observed frequencies of strength deficit. Furthermore, the Kolmogorov-Smirnov (KS) was employed for assessing normality of the distribution of scores. The Levene’s test was used to assess homogeneity of variance between groups. A significant level was accepted at the 95% confidence level for all statistical parameters (p<0.05).

## Results

### Comparison of PT between dominant and non-dominant legs

Means and SD of peak torque of the hamstring and quadriceps muscle groups in the dominant and non-dominant leg are presented in Table 1. The PT of both muscles in the non-dominant leg at all angular velocities showed higher tendency than the dominant leg. However, no significant differences were found between the dominant and non-dominant legs in the PT of quadriceps muscles (F_1,35_=1.05, p=0.311) nor hamstring muscle (F_1,35_=1.63, p=0.209). Repeated measures ANOVA results showed significant differences of PT among three angular velocities in the quadriceps (F_2,34_=176.4, p=0.001) and hamstring muscles (F_2,34_=48.3, p=0.001).

### Comparison of deficit between dominant and non-dominant legs

The profile of deficits in the quadriceps and hamstring muscles is shown in Table 2. Deficits were abnormal at all angular velocities (more than 10% difference). Only one player was found normal at all angular velocities of knee muscles, but 35 players (97.2%) were found to have at least one musculoskeletal abnormality of more than 10% at one or more angular velocities. *Chi*-square showed a significant difference only in hamstring muscles at 180°/s angular velocity (p=0.008).

### Comparison of strength ratio between dominant and non-dominant legs

No significant difference (p>0.05) was found between dominant and non-dominant legs in CSR, DCR and F/S ratio (muscles peak torque at 60°/s / muscles peak torque at 300°/s). The profile of the strength ratio is shown in Table 3. There was a significant difference between angular velocities in CSR (F_2,34_=22.9, p=0.01). In all cases PT were highest at slow velocities (60°/s), followed by medium velocities (180°/s), and lowest at high velocities (300°/s).

### Hip joint flexibility between dominant and non-dominant legs

**Figure 1:**
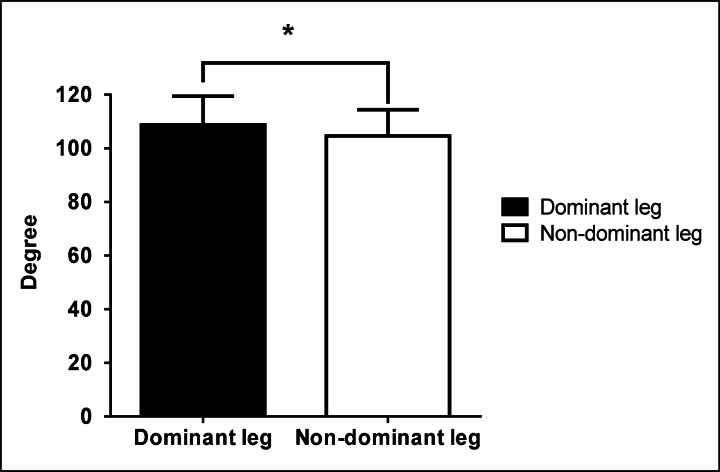
Flexibility in the dominant and non-dominant leg. Higher degree of flexibility is shown in the dominant leg (*p<0.05).

A significant difference was revealed (t=4.7, p=0.001) between the mean hip joint flexibility of the dominant and non-dominant legs (108.8 ± 10.7° versus 104.6± 9.8°, respectively). In this case the flexibility in the non-dominant leg was lower than that of the dominant leg (Figure 1).

## Discussion

The aim of this study was to investigate the bilateral and unilateral asymmetries of strength and flexibility in young male professional soccer players. Peak torques in the non-dominant leg at all angular velocities seemed higher than the dominant leg, however, no significant differences were revealed. These results are in agreement with the study of Rahnama et al. (2005) that reported no significant differences between the two legs in knee extensors at three different velocities (60, 120, 300°/s) among elite soccer players. Tourny-Chollet et al. (2000) investigated 21 (22.0±2.95 years) amateur female soccer players. Their results showed a significant difference at medium speed between hamstring muscles in the dominant and non-dominant legs. Tourny-Chollet et al. (2000) concluded that the knee flexor of the dominant leg tended to be stronger than that of the non-dominant leg. The finding is, however, in contrast with that of the present study. These conflicting results may be caused by the differences in subjects’ characteristics such as the level of play, age and gender. Gender-related factors include anatomy, hormonal profile, ligament laxity, and the effect of menstrual cycles on the knee strength. Typically women have a laxity of the ligaments around the knee joint (Loes et al., 2000; Dugan, 2005) which may impact upon knee strength.

The results showed that strength deficits were abnormal (more than 10%) at all angular velocities. The strength deficit is defined as the difference between the strength of muscles of opposite extremities. These results are parallel with those studies that found a majority of soccer players have bilateral strength differences (Ekstrand and Gillquist, 1982; Chin et al., 1994; Rahnama et al., 2005). The strength deficit of more than 10% contributes to a knee risk factor (Fousekis et al., 2010; Brito et al., 2010). In a sport with asymmetric kinetic patterns, more emphasis is given to one side of the legs (Schiltz et al., 2009). According to Iga et al. (2009), soccer players almost never use both legs with equal emphasis. Soccer players’ preference to use one side more than the other is related to hemispheric dominance of the brain in the opposite side. This is the possible cause for the deficit abnormality in professional soccer players.

No difference was observed in CSR, DCR and F/S speed ratios between the dominant and non-dominant legs, although the non-dominant leg seemed to have higher ratios. Brito et al. (2010) studied isokinetic knee strength ratio in sub-elite male soccer players, and their results showed that the ratio in the non-dominant leg is more than that in the dominant leg. They found that hamstrings’ peak torque of the non-dominant leg is stronger than in those of the dominant leg (Brito et al., 2010). The conventional strength ratio is calculated as peak torque of hamstrings divided by that of quadriceps muscles. Increasing flexor PT improves the knee strength ratio at this angular velocity. These conflicting results may be caused by the differences in the level of play of the subjects. Professional soccer players have higher quadriceps strength than non-professional players (Gil et al., 2010).

The results of the present study showed the CSR (normal average; 0.61, 0.72, 0.78 at 60°/s, 180°/s and 300°/s, respectively) and DCR (normal average>1.0) of the players were below the normal average values at various angular speeds which predispose the players to knee injuries. Kim and Hong (2011) studied the National College American Association (NCAA) athletes and found an association of lower than 0.6 of the CSR at 60°/s and non-contact leg injuries, suggesting the significant contribution of hamstring to quadriceps imbalance to non-contact leg injuries. Fousekis et al. (2011) measured intrinsic risk factors during pre-season in 100 professional soccer players and found players with eccentric hamstring strength asymmetries were at greater risk of hamstring strain while players with eccentric strength and flexibility asymmetries in their quadriceps were at greater risk of quadriceps strain. The anterior cruciate ligament (ACL) and hamstring become more susceptible to injury with a mismatch of hamstring to the quadriceps strength ratio. This is because the strength of hamstring is protective against anterior translation of the tibia on femur which occurs during landings and sudden changes in direction. A lower hamstring to quadriceps strength ratio allows higher shear forces on the ACL during these activities. Furthermore, hamstrings strain which commonly follows eccentric lengthening during the terminal swing can be attenuated by increasing the hamstrings strength (Holcomb et al., 2007).

In this study we found that the hip flexibility in the dominant leg was significantly higher than that in the non-dominant leg. The dominant leg is used to handle an object or to lead out, while the non-dominant leg has the main role of providing postural support. This definition of footedness is commonly accepted by researchers (Hardt et al., 2009). Soccer players use one favoured foot unilaterally for kicking the ball (Rahnama et al., 2005; Fousekis et al., 2010). This preference is a possible cause of an asymmetry in flexibility. Professional soccer players can perform a higher Dynamic Range of Motion of the hip joint during an instep kick after dynamic stretching (Amiri-Khorasani et al., 2011). Kicking by the dominant leg is repeated many times in soccer and it is the same as dynamic stretching that may be caused by higher flexibility in the dominant leg. However, we found that the non-dominant leg has lower flexibility which may lead to injury. Gabbe et al. (2005) showed that decrement in quadriceps flexibility is a predictor to hamstring injury. Worrel et al. (1991) found that the hamstring in the injured group was less flexible than the non-injured group, and that the injured leg was less flexible than the bilateral side. It should be noted that the injured group recruited in his study were those with history of hamstring injury. It is known that hamstring tightness is a risk factor to hamstring injury (Verrall et al., 2001).

There were significant differences between different angular velocities of knee muscles in the dominant and non-dominant legs. The result indicated that the high PT was at the slow speed (60°/s) and the low PT at the higher speed (300°/s). These results are in agreement with Kofotolis and Kellis’ (2007) findings. Increasing angular velocity had a significant effect on the PT in both legs. Hill’s curve explaining force-velocity showed that muscle fibres produce less contraction while the speed of contraction increases (Malý et al., 2010). The time available for contact between actin and myosin filaments decreases with increasing velocity of concentric activity (Huxley model); thus, period of the contact phase reduces in the overall cycle. Cross-bridges have to be re-released shortly after their connection without enough time to produce power, so the proportion of combined bridges in the muscle declines, and produces lower strength (Malý et al., 2010). Compressive and translation forces across a joint vary with speed. Slower speeds have more compressive force than faster speeds. More motor units can be recruited at slower speeds than higher speeds allowing more torque production (Bottinelli et al., 1996).

## Conclusion

Soccer players usually have some level of musculoskeletal abnormalities and professional soccer training should aim to improve the flexibility of both dominant and non-dominant legs. It is common that the non-dominant leg is less flexible compared to the dominant leg; hence, more emphasis and vigilance should be directed at more balance flexibility. The findings of this research can be helpful for coaches and trainers who can strategize training programs to improve balance and strength of young professional soccer players. The results of the present study support the fact that professional soccer training programmes and competitions at professional levels can affect the strength of the knee joint muscles. These results affirm the possibility that the unique muscle-loading patterns experienced in soccer over time, together with the superior use of the dominant leg, may result in the bilateral asymmetrical increase of the concentric strength of the quadriceps muscles in the dominant kicking leg. The present findings reveal that physical performance and movement pattern experienced during soccer may negatively change the bilateral deficit, but do not have significant effect on the PT, CSR and DCR.

## Figures and Tables

**Table 1:** Isokinetic peak torque in quadriceps and hamstring muscles (values are mean ± SD), and percentage of change (Δ) {values are mean (95% CI)} of dominant to non-dominant legs.

	Dominant (Nm)	Non-dominant (Nm)	Δ% (95% CI)	p
Q_Con_60 °/s	201.8 ± 47.8	209.3 ± 50.5	−7.5(−17.9 to 2.9)	
Q_Con_180°/s	129.2 ± 37.9	129.8 ± 37.2	−0.64(−8.2 to 6.2)	p= 0.311
Q_Con_300°/s	93.2 ± 30.6	95.8 ± 30.2	−2.6(−8.5 to 3.2)	
H_Con_60°/s	100.1 ± 27.6	102.3 ± 30.5	−2.1(−10.7 to 6.4)	
H_Con_180°/s	65.5 ± 25.6	70.8 ± 21.1	−5.3(−10.2 to −0.42)	p= 0.209
H_Con_300°/s	66.8 ± 25.1	67.9 ± 24.8	−1.1(−5.7 to 3.4)	

Q = Quadriceps muscles; H = Hamstring muscles; Con = concentric; Nm = Newton meter; °/s = degree per second; CI= confidence interval.

**Table 2:** Isokinetic deficit (values are mean ± SD) in quadriceps and hamstring muscles (statistical values are Chi-square and p-value).

		Normal		Abnormal		

	Deficit (Nm)	Frequency	Percent (%)	Frequency	Percent (%)	χ^2^ p
Q_Con_60°/s	13.8 ± 14.6	20	55.6	16	44.4	χ^2^ =0.44, p= 0.505
Q_Con_180°/s	15 ± 16	20	55.6	16	44.4	χ^2^ =0.44, p= 0.505
Q_Con_300°/s	16.8 ± 16.5	17	47.2	19	52.8	χ^2^ =0.11, p= 0.739
H_Con_60°/s	19.6 ± 30.3	15	41.7	21	58.3	χ^2^ =1.00, p= 0.317
H_Con_180°/s	21.8 ± 19.7	10	27.8	26	72.2	χ^2^ =7.11, p= 0.008*
H_Con_300°/s	15.2 ± 11	15	41.7	21	58.3	χ^2^ =1.00, p= 0.317

Q= Quadriceps muscles; H = Hamstring muscles; Con= concentric; Nm= Newton meter; °/s = degree per second; χ^2^ = Chi-square;

* p<0.05.

**Table 3:** Conventional and dynamic control ratio in dominant and non-dominant legs (values are mean ± SD), and percentage of change (Δ) (95% CI) of dominant to non-dominant legs.

	Dominant	Non-dominant	Δ% (95% CI)	p-value
**Conventional ratio**				
H/Q _Con_ 60°/s	0.50 ± 0.11	0.50 ± 0.14	0.001(−0.04 to 0.0 5)	
H/Q _Con_ 180°/s	0.51 ± 0.13	0.56 ± 0.14	−0.05(−0.09 to −0.009)	p= 0.30
H/Q _Con_ 300°/s	0.74 ± 0.22	0.75 ± 0.26	−0.001(−0.05 to 0.04)	
**Fast/Slow speed ratio**				
F/S Quadriceps	2.3 ± 0.9	2.4 ± 1.3	−0.10(−0.3 to 0.9)	p= 0.36
F/S Hamstring	1.6 ± 0.7	1.7 ± 1.1	−0.08(−0.4 to 0.2)	p= 0.53
**Dynamic control ratio**				
H_ECC_/Q_CON_ 120°/s	0.74 ± 0.35	0.75 ± 0.33	−0.01(−0.11 to 0.10)	p= 0.81

Conventional ratio (concentric knee flexion / concentric knee extension); F/S =Fast/Slow speed ratio (muscles peak torque at 60°/s / muscles peak torque at 300°/s); Dynamic control ratio= eccentric PT of hamstrings / concentric PT of quadriceps; Q= Quadriceps muscles; H= Hamstring muscles; Con=concentric; Nm= Newton meter; °/s= degree per second.
